# Prophylactic Cranial Irradiation in Small Cell Lung Cancer: Evolution of Evidence, Current Status, and Future Directions

**DOI:** 10.3390/cimb47120998

**Published:** 2025-11-28

**Authors:** Swati Mamidanna, Menal Bhandari, Charvi Shah, Ludvinna Bazile, Sukhdeep Kaur Gill, Adeel Riaz, Lakshmi Rekha Narra, Shreel Parikh, Ahmed Shalaby, Mihir Patel, Zohaib Khan Sherwani, Jongmyung Kim, Matthew P. Deek, Salma K. Jabbour, Ritesh Kumar

**Affiliations:** 1 Department of Radiation Oncology, Rutgers Cancer Institute, Rutgers University, New Brunswick, NJ 08901, USA; sm2293@cinj.rutgers.edu (S.M.); ar2354@rwjms.rutgers.edu (A.R.); ln303@rwjms.rutgers.edu (L.R.N.); shreel.parikh@rutgers.edu (S.P.); as4198@cinj.rutgers.edu (A.S.); m.patel0407@rutgers.edu (M.P.); sz698@cinj.rutgers.edu (Z.K.S.); deekmp@cinj.rutgers.edu (M.P.D.); jabbousk@cinj.rutgers.edu (S.K.J.); 2 Department of Radiation Oncology, Loyola University, Chicago, IL 60660, USA; menalbhandari18@gmail.com; 3 Robert Wood Johnson Medical School, Rutgers University, New Brunswick, NJ 08901, USA; ccs136@rwjms.rutgers.edu (C.S.); lb773@njms.rutgers.edu (L.B.); 4 Department of Radiation Medicine, University of Kentucky, Lexington, KY 40536, USA; sukhdeep.gill@uky.edu; 5 Department of Radiation Oncology, Moffitt Cancer Center, Tampa, FL 33612, USA; jongmyung.kim@moffitt.org

**Keywords:** small-cell lung cancer, prophylactic cranial irradiation, neurocognition

## Abstract

Small cell lung cancer (SCLC) is an aggressive malignancy with a high incidence of brain metastases. Prophylactic cranial irradiation (PCI) was developed to reduce central nervous system (CNS) relapses and has been shown to improve survival, particularly in limited-stage disease. The pivotal Auperin meta-analysis and subsequent studies confirmed its role in patients achieving a complete response to initial therapy. In extensive-stage SCLC, earlier trials demonstrated reduced brain metastases and modest survival gains, but more recent studies incorporating routine magnetic resonance imaging (MRI) surveillance failed to show overall survival benefits, supporting MRI monitoring with salvage therapy as an alternative. Neurocognitive toxicity remains the major limitation of PCI, especially in older adults. Common effects include memory impairment, cognitive changes, and a reduced quality of life. Advances such as hippocampal avoidance PCI and neuroprotective strategies like memantine have shown the ability to mitigate long-term decline. Modern radiotherapy techniques, including intensity modulated radiation therapy (IMRT) and volumetric modulated arc therapy (VMAT), enable the precise sparing of critical structures while maintaining intracranial control. The integration of immunotherapy has shifted treatment paradigms in SCLC. While checkpoint inhibitors have improved systemic outcomes, their impact on brain relapses and interactions with PCI remain uncertain. This review provides an overview of the evolution of PCI in SCLC, while emphasizing current challenges and future directions.

## 1. Introduction

Small cell lung cancer (SCLC) is a highly aggressive neuroendocrine malignancy strongly associated with tobacco exposure, accounting for approximately 15% of all lung cancers [1]. Despite the high initial responsiveness to treatments, including chemoradiotherapy, chemotherapy alone, chemoimmunotherapy, or chemoradiotherapy along with immunotherapy, the overall prognosis remains poor, with a median survival of less than two years in limited-stage (LS-SCLC) and about one year in extensive-stage disease (ES-SCLC). SCLC is characterized by rapid proliferation, early dissemination, and a strikingly high incidence of brain metastases, observed in up to 10% of patients at diagnosis and an additional 40–50% over the disease course. This proclivity for central nervous system (CNS) involvement is thought to result from the high burden of circulating tumor cells, its tendency for hematogenous spread, and the concept that the brain is a sanctuary site not well penetrated by traditional chemotherapy. Therefore, patients have the tendency to relapse in the brain [2]. To address this, prophylactic cranial irradiation (PCI) was introduced and developed as a preventive strategy aimed at eradicating micro-metastatic disease in the brain, and early studies demonstrated a significant reduction in CNS relapses and overall survival benefits in selected patients.

However, the role of PCI in the modern era has become increasingly nuanced. Concerns surrounding neurotoxicity, particularly in older adults and those with preexisting cognitive deficits, have prompted a re-evaluation of its use. Evolving imaging techniques, particularly high-resolution magnetic resonance imaging (MRI), now allow for the earlier detection and management of asymptomatic brain metastases, offering an alternative approach of active surveillance with deferred stereotactic radiosurgery (SRS) or whole-brain radiation therapy (WBRT) upon the detection of brain metastasis. Moreover, contemporary trials have failed to show a clear survival benefit of PCI in ES-SCLC in the context of regular MRI monitoring, further complicating its routine use. The introduction of immunotherapy in the management of SCLC has led to a new enthusiasm in the treatment paradigm of SCLC, but at the same time it is gradually shifting treatment focus away from PCI. The variability in international guidelines also reflects this uncertainty, with differing recommendations from ASTRO, ESTRO, and NCCN based on the disease stage, patient performance status, and neurocognitive risk (Table 1). Recent innovations such as hippocampal-sparing PCI (HA-PCI) and pharmacologic neuroprotectants are being explored to mitigate long-term neurocognitive decline while preserving oncologic efficacy.

## 2. Evolution or Rationale of PCI in SCLC

Many early studies in patients with SCLC performed in the 1970s–1980s showed that up to 50% of patients had CNS metastatic disease at the time of autopsy, and typically this was multiple metastases and contributed as a major cause of death. Originally, brain radiation was thought of as a palliative measure to improve the neurological consequences of CNS disease burden. Many of these early trials included prophylactic radiation to the brain in the upfront setting without an adequate control of local/regional disease, and there was typically no benefit to survival, likely due to death from extracranial systemic disease. However, there were reports of improved neurologic function because of decreased brain metastases due to PCI [6]. Several trials showed clinically meaningful significant benefits of decreasing relapses in the CNS [7,8,9].

### 2.1. PCI in LS-SCLC

For stage I patients who are undergoing surgery, the role of PCI is ill defined. A meta-analysis of 1691 patients by Yang et al. revealed a reduced risk of brain metastasis and improved overall survival with PCI after a resection of the primary disease. However, in a subgroup analysis, they revealed that the risk of brain metastasis is relatively low, about 12% for stage I SCLC patients, with no survival benefit in those patients with PCI (HR:0.87, 95% CI:0.34 to 2.24) [10].

Initial studies for PCI in LS-SCLC primarily incorporated patients who achieved very good responses/complete clinical and radiographic response to treatment. Arguably the most critical study investigating the role of PCI in LS-SCLC is the Auperin meta-analysis [11]. Published in 1999, this study combined data from seven trials of patients with SCLC, for a total of 987 patients. The primary endpoint of the meta-analysis was to ascertain if PCI improves survival. After receiving an initial treatment with chemotherapy ± radiotherapy, those patients that achieved a complete response were randomized to receive PCI or no PCI. The meta-analysis found that there was a statistically significant absolute benefit to overall survival of 5.4% at the 3-year follow-up (15.3% vs. 20.7%), correlating with the relative risk of death being 0.84 (95% CI, 0.73 to 0.97; *p* = 0.01) in those that received PCI. The cumulative incidence of brain metastases also significantly decreased by 25.3% in the PCI group (58.6% vs. 33.3%), correlating to a relative risk of 0.46 (95% CI, 0.38 to 0.57; *p* < 0.001). The subgroup analysis also showed a trend toward fewer brain metastases in those patients that were treated sooner after induction therapy, but with no difference in the overall survival. Early PCI studies were performed with less sensitive imaging, such as chest X-rays (CXRs), for response assessment, and some used CT of the brain, while others did not systematically image the brain. Therefore, before the use of modern imaging and more rigorous staging, there may have been a cohort of patients with undetected brain metastases who benefitted from treating the existing disease, leading to an inflation of the benefits of PCI.

This meta-analysis was conducted more than two decades ago, and therefore there are multiple aspects of this analysis that should be taken into consideration. Some trials included in the meta-analysis did not require imaging of the brain. The dose, fractionation, and time from initial therapy to PCI was also not consistent between studies. Ultimately, following publication, this meta-analysis established PCI as a standard of care treatment in patients with LS-SCLC who had a complete response to initial therapy.

More recently, Tomassen et al. conducted a meta-analysis to learn more about the role of PCI in LS-SCLC in the present era [12]. After analyzing more than 18,000 patients from 28 retrospective studies, they demonstrated that patients with LS-SCLC who undergo PCI have a significantly better OS, with an HR of 0.62 (95% CI 0.57–0.69). Also, they found out that the effect of PCI was notable and significant whether the studies were conducted before 2018 or after 2018. So, the authors concluded that, despite ongoing research, the evidence suggests that PCI remains a standard treatment for patients with LS-SCLC seeking to improve overall survival. However, this meta-analysis relies on retrospective data, and therefore there are some limitations in the conclusions that can be drawn from the study.

### 2.2. PCI in ES-SCLC

The role of PCI in ES-SCLC is more nuanced compared to its role in LS-SCLC. In 2007, Slotman et al. published their trial evaluating patients with ES-SCLC who were randomized to receive PCI or no PCI [13]. Eligible patients were those who had any response to chemotherapy, no evidence of brain or leptomeningeal metastasis, and a good performance status (ECOG 0–2). Patients were considered to have ES-SCLC if they were found to have disease outside of the ipsilateral hemithorax, ipsilateral supraclavicular nodes, or malignant pleural effusion. A total of 286 patients were randomized, with the experimental arm receiving PCI ranging from 20 to 30 Gy in 5–12 fractions (20 Gy in 5 fractions was the most common regimen), and the primary endpoint was time to symptomatic brain metastasis. The results at 1 year demonstrated that PCI improved the time to symptomatic brain metastasis (14.6% vs. 40.4%). The overall survival rate at 1 year also benefited the PCI arm (27.1% vs. 13.3%), correlating to a median overall survival time of 6.7 months vs. 5.4 months. These results indicate a significant benefit from the use of PCI in the ES-SCLC population. Nevertheless, there are some limitations of this study. Imaging of the brain was not necessitated for staging or follow-up in the absence of symptoms, suggesting cranial metastasis, possibly allowing patients with intracranial metastases to go undetected. There was no standardized radiation dose or fractionation scheme, no information regarding chemotherapy regimens provided, and no standardized criteria defining treatment response.

Takahashi et al., who published their findings in 2017, assessed the use of PCI in ES-SCLC, comparing it specifically to a regimented surveillance approach, but with the intent of clarifying the findings of prior studies and accounting for their limitations [14]. ES-SCLC patients with a good performance status (ECOG 0-2), any treatment response to platinum doublet therapy, and no brain metastases confirmed with a baseline MRI were included. A total of 224 patients were enrolled, with 113 randomized to PCI with 25 Gy in 10 fractions and 111 patients to the observation arm, with a primary endpoint of overall survival. MRI surveillance was required every 3 months for the first year, then every 6 months for the next year. The results at 1 year showed no significant difference in the medial overall survival between arms (11.6 months in PCI arm vs. 13.7 months in observation arm, *p* = 0.094). There was a reduction in the overall occurrence of brain metastases, as the PCI arm had only 48% of patients develop brain mets compared to 69% of patients in the observation arm, but no difference was seen in cognitive functioning as assessed using the MMSE. At the interim analysis, based upon the very low predictive probability of PCI showing significant benefits compared to observation, this trial was closed early. These results thus support that regular monitoring with surveillance MRI is a viable alternative to PCI in the setting of ES-SCLC. One possible explanation of the lack of significant benefits of PCI is the routine use of MRI brain surveillance in the above study, whereas the survival benefit observed in earlier trials may have reflected a therapeutic rather than prophylactic effect of PCI. While the Slotman study was positive for survival, in general, both studies had relatively small sample sizes.

## 3. RT Dose Evolution

PCI dosing has evolved as studies have sought to balance achieving a reduced incidence of brain metastases with acceptable treatment-related toxicities. An early study by Suwinski R et al. reported a dose–response relationship with a radiation dose of 30–35 Gy when given immediately after chemotherapy and showed a reduction in the incidence of brain metastases; higher doses did not provide additional benefits [15]. This dose–response relationship was also observed in the Auperin meta-analysis of seven randomized control trials evaluating PCI [11]. These studies used a range of regimens, including 24 Gy in 8 fractions, 25 Gy in 10 fractions, 30 Gy in 10 fractions, 36 Gy in 18 fractions, and 40 Gy in 20 fractions [16,17,18,19,20]. When these regimens were grouped by total dose (8 Gy, 24–25 Gy, 30 Gy, and 36–40 Gy), a significant trend towards lower rates of brain metastases was seen with higher doses (*p* = 0.02), which supports the dose–response effect for intracranial control. Another randomized trial performed in 6 centers from the UK and 10 centers from the EORTC also allowed a wide variation in doses, from 20 to 36 Gy, and also reported that higher doses of 36 Gy were more effective in reducing the risk of brain metastases compared to those treated with 24 Gy.

To further clarify the clinical relevance of this effect, RTOG 0212 has compared standard-dose vs. high-dose PCI in patients with limited-stage SCLC [21,22]. Published in 2009 with a further subset analysis published in 2011, this trial compared the standard dose of 25 Gy in 10 once-daily fractions to either 36 Gy in 18 once-daily fractions or 36 Gy in 24 twice-daily fractions [23]. Patients were eligible if they demonstrated a complete response after initial chemotherapy and thoracic radiotherapy, with a minimum requirement of a normal chest X-ray. Imaging of the brain was performed within 1 month of randomization with either CT or MRI and required no evidence of metastasis in the brain or leptomeninges. A total of 720 patients were randomized, with the primary endpoint being the incidence of brain metastases at 2 years. The results at 2 years demonstrated no statistical difference in the incidence of brain metastases (29% standard arm vs. 23% higher-dose arm, *p* = 0.18). The overall survival showed a trend towards worse outcomes for the high dose arm (42% standard arm vs. 37% higher dose arm, *p* = 0.05), and no significant differences were seen in the acute toxicity between groups. At a 3-year follow-up, there remained no significant difference between groups in neurocognitive function. This study reaffirmed the standard dose for PCI as 25 Gy in 10 once-daily fractions. A further analysis of the RTOG 0212 patients by Wolfson et al. in 2011 showed a significant increase in chronic neurotoxicity in the 36 Gy arm (*p* = 0.02), and also demonstrated that increases in age are the greatest predictor of the development of chronic neurotoxicity [22].

On the other hand, in ES-SCLC, two key trials have evaluated the role of PCI using different doses and fractionation strategies. Slotman et al. allowed a range of regimens, including 20 Gy in 5 or 8 fractions, 24 Gy in 12 fractions, 25 Gy in 10 fractions, and 30 Gy in 10 or 12 fractions, corresponding to BEDs of 28 to 39 Gy (using an α/β ratio of 10 Gy) [13]. The most used regimen was 20 Gy in 5 fractions. This trial demonstrated a significant reduction in brain metastases and an improvement in overall survival. In contrast, in 2017, Takahashi et al. allowed only the standard 25 Gy in 10 fractions, which showed a reduced incidence of brain metastases with no survival benefits [14]. Given the limited prognosis in extensive-stage disease, shorter regimens such as 20 Gy in 5 fractions as commonly used in Slotman et al. may be an alternative option compared to the standard 25 Gy in 10 fractions. Nevertheless, the standard of care for both limited- and extensive-stage SCLC for PCI is 25 Gy in 10 fractions.

## 4. Hippocampal Avoidance (HA) WBRT in SCLC

PCI is an established standard of care for LS-SCLC with some debate in the ES-SCLC because of neurotoxic concerns. Several studies have demonstrated neurocognitive decline, memory loss, and a poor quality of life in patients treated with WBRT. Definitions of neurotoxicity vary, but several studies show that memory impairment is seen, especially in immediate and delayed recall and worse self-perceived cognition [24,25]. The severe memory loss and intellectual decline may contribute to an impaired quality of life. Slotman et al. showed that health-related quality of life (HRQOL) was worse in SCLC patients treated with PCI vs. the observation [26]. Le Pechoux et al. demonstrated neurocognitive decline with WBRT, but there was no impact of different dose levels on neurocognitive decline [23]. The hippocampus is a vital brain structure involved in memory, learning, spatial processing, and emotional regulation. Studies have shown that cranial irradiation affects the progenitor cells in the hippocampi, causing alterations of their mitotic activity, differentiation, and neurogenic signaling, which might be the reason for the changes in cognitive function following radiation treatment [27]. Neurocognitive decline from cranial irradiation as a byproduct of hippocampal irradiation was further strengthened from a dosimetry study where the dose to the hippocampi was found to be associated with a decline in long-term learning [27].

This emerging evidence and better radiation therapy delivery techniques, like intensity modulated radiation therapy (IMRT) and volumetric modulated arc therapy (VMAT), led to the concept of WBRT with hippocampal avoidance (HA-WBRT) for preserving neurocognitive function, where hippocampi can be easily spared, with a minimal impact of dose to the remaining whole brain. Radiation Therapy Oncology Group (RTOG) 0933 is a phase II study of HA-WBRT in patients with brain metastases, which showed that HA-WBRT is feasible and significantly improves memory function preservation [28]. NRG-CC001 is a phase III trial in patients with brain metastases comparing WBRT plus memantine vs. HA-WBRT plus memantine, demonstrating that cognitive function and patient-reported symptoms are better preserved with HA-WBRT plus memantine, with no difference in intracranial recurrence [29].

After demonstrating the benefits to neurocognitive function of HA-WBRT in multiple randomized studies for brain metastasis from other primary malignancies, the next question is if HA-WBRT can be safely performed in SCLC for PCI with the same neurocognitive protection and equivalent intracranial recurrence rates. Also, although HA-WBRT has been primarily evaluated in patients with intact brain metastases, its use in prophylactic cranial irradiation (PCI) for small cell lung cancer (SCLC) has been limited due to the presumed risk of disease relapse within the hippocampal and perihippocampal regions, as defined by the RTOG 0933 atlas [28]. Earlier studies exploring the patterns of failure have reported a low incidence of recurrence in these regions [30,31,32]. Kundapur et al. demonstrated that only 2.2% of lesions were in the hippocampus proper and another 2.2% were within the 5 mm perihippocampal ring, and the authors cautioned that patients with larger hippocampal volumes and many brain metastases might be poor candidates for HA-WBRT because their risk of harboring lesions in that region is higher [32]. A recent study by Cook TA et al. has shown that 3 of 17 patients who received HA-PCI had a multifocal relapse of the disease which included the hippocampal avoidance zone [33]. The PREMER trial randomized 150 SCLC patients for PCI (25 Gy in 10 fractions) with or without HA [31]. With a median follow-up of 40.4 months, the primary end point of delayed free recall (DFR) on the Free and Cued Selective Reminding Test (FCSRT) at 3 months was lower in HA-PCI compared to PCI (5.8% vs. 23.5%, OR 5, 95% CI 1.57–15.86, *p* = 0.003). The incidence of brain metastasis and overall survival were not significantly different in either of the arms. Belderbos et al. conducted another phase III study (NCT0178067) similar to the PREMER trial randomizing 168 SCLC patients to PCI with or without HA [34]. At a median follow-up of 26.6 months, the primary endpoint of the Hopkins Verbal Learning Test Revised (HVLT-R) delayed at 4 months was similar in HA-PCI vs. PCI (28% vs. 29%, *p* = 1.0). Brain metastasis and overall survival were also similar in both arms.

The NRG CC003 phase II/III trial randomized 392 SCLC patients to PCI or HA-PCI [35]. At a median follow-up of 14.9 months, the intracranial recurrence rate was similar in both arms (14.8% vs. 14.2%, *p* = 0.0001). The primary end point of HVLT-R delayed recall at 6 months was not significantly different (PCI 30.0% vs. HA-PCI 26.0%, *p* = 0.31); however, neurocognitive function failure was better with HA (adjusted HR = 0.77, 95% CI: 0.61–0.98, *p* = 0.03). In summary, studies have shown that HA WBRT is safe in SCLC, with a similar intracranial recurrence rate as compared to WBRT in carefully selected patients. Two of the above-mentioned studies demonstrated significant benefits in cognitive function after HA-WBRT, so this approach of HA can be considered for PCI as well.

## 5. RT Planning and Dose Constraints

### 5.1. WBRT

**Figure 1:**
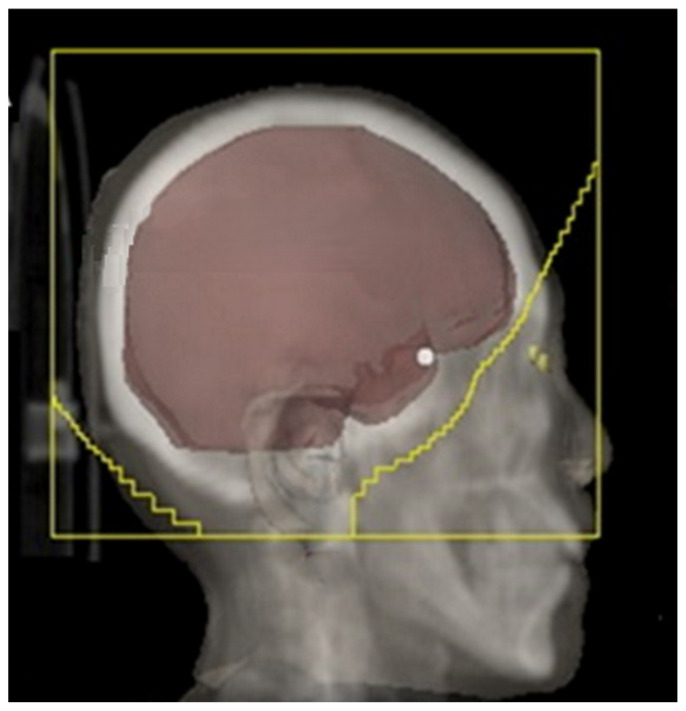
WBRT fields (adapted from Figure 1A from [38]).

For conventional whole-brain radiotherapy (WBRT) using a three-dimensional conformal technique, patients are simulated in the supine position with immobilization provided by a thermoplastic mask. A non-contrast planning CT scan is obtained from the vertex to the mid-thoracic spine using a regular 2–3 mm slice thickness. WBRT is ideally planned by using opposed lateral fields with high energy megavoltage photons and the dose is prescribed to the midline. Field borders should include a 1–2 cm flash in the superior, anterior, and posterior borders of the skull. While designing the inferior border, one must ensure a 0.5 to 1 cm margin below the cribriform plate, covering the floor of the middle cranial fossa and extending into the inferior end plate of the C2 vertebra [Figure 1]. The clinical target volume would include the whole brain using the inner table of the skull, outlined using the bone window setting including the whole frontal lobe, temporal lobes, pituitary fossa, and cribriform plate. Attention can be paid to the parotid gland dose when extending this field to C2 [36,37].

### 5.2. IMRT Planning

**Figure 2:**
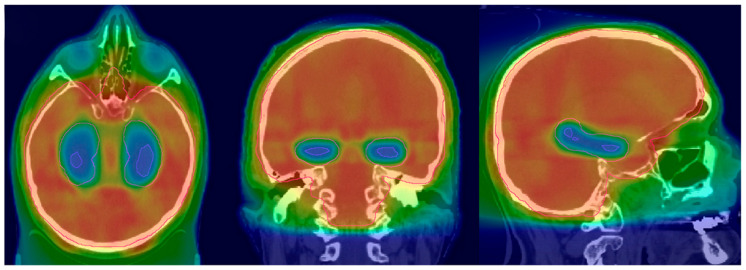
HA-PCI dose distribution (adapted from Figure 1A from [39]).

IMRT is the preferred technique for HA-PCI. The CT simulation process is similar to the 3D-WBRT, with patients positioned supine and immobilized using a thermoplastic mask. However, a thinner slice thickness, typically between 1.25 and 1.5 mm, is preferred, as it allows for precise hippocampal contouring. Prior to the planning, thin slice brain MRI images fused with a CT simulation scan are preferred for hippocampal contouring. Axial T1-weighted with contrast and T2-weighted images are used for the delineation of hippocampi and other potential OARs. Specific to HA-PCI, the planning target volume (PTV) is the whole brain minus the hippocampal avoidance zone (bilateral hippocampi and a 5 mm 3D expansion around it), per the RTOG 0933 atlas [28]. While planning HA-PCI, one must make sure to minimize the dose to the hippocampi while achieving an adequate PTV coverage. The goal is to cover 95% of the PTV to a 100% prescription dose [Figure 2].

### 5.3. Constraints

IMRT is widely used in PCI planning due to its ability to generate conformal dose distributions and reduce doses to surrounding normal tissues [40]. Dose-dependent toxicities have been reported with higher PCI doses, at 30 to 36 Gy [40,41,42]. Pituitary dysfunction is one such effect, with TSH suppression reported in up to 30% of patients, which may remain altered in the long term in approximately 9% of patients. If left untreated, this can lead to secondary hypothyroidism, which can superimpose cognitive and existing neuropsychiatric symptoms [43]. The risk of hypopituitarism increases in a dose-dependent manner after exceeding 30 Gy. Moreover, alopecia is a common acute side effect of cranial irradiation. IMRT has been shown to reduce the scalp dose; a study by Roberge D et al. has demonstrated a 40% reduction in the median scalp dose without compromising target coverage [44]. While this may not fully prevent transient alopecia, it may offer benefits particularly in high-risk patients. For hippocampal avoidance WBRT, the most validated constraints are referred to as D100% ≤ 9 Gy and Dmax ≤ 16 Gy to the hippocampi [29]. Additional organs at risk include the scalp, with a target OAR dose (mean < 18 Gy) and parotids V20 of Gy < 47%, and the cochlea, with a mean < 22 Gy [45,46]. The constraints for the optic structures such as the nerves and chiasm are Dmax < 30 Gy.

## 6. Role of PCI in Immunotherapy Era

As detailed above, earlier studies have shown that, in patients with SCLC, PCI lowers the risk of brain metastases and provides a survival benefit and hence has been incorporated into the standard of care. However, the limited long-term success of these treatments associated with disease relapse or drug resistance, given the unique tumor biology of small cell lung cancer and high mutational burden, has led to an increased interest in immunotherapy (IO). Table 2 reviews the various immunotherapy agents that have made breakthroughs, shifting treatment paradigms over the past decade.

In this current era of immunotherapy, the role and importance of PCI are still evolving, with the risks and benefits of combining IO with PCI necessitating cautious interpretation. While PCI has been shown to reduce the risk of brain metastases, the potential for IO agents to cross the blood–brain barrier (BBB) is still unclear. Tumor cells that metastasize to the brain have a mechanism to evade the immune system [47]. There are no clear pharmacokinetic or pharmacodynamic studies which have confirmed that immune checkpoint inhibitors can penetrate the BBB [48]. However, studies show that, rather than permeating the CSF, one method of action for checkpoint inhibitors is peripherally through immunogenic cell death by the stimulation of T cells, allowing them to enter the central nervous system to produce anti-tumor effects, leading to an abscopal effect [49].

PCI may increase PD-L1 expression, making tumor cells more susceptible to checkpoint inhibitors, leading to a potential synergistic effect with immunotherapy. On the other hand, the potential risk of combining PCI with IO is that, as IO can cause systemic inflammation, the toxicities of neurocognitive impairment may be amplified.

The landmark trials in ES-SCLC, such as IMPower133, CASPIAN, Keynote604, and Capstone-01, have all tested first-line regimens of various immune checkpoint inhibitors in combination with chemotherapy and yielded improved survival outcomes [50,51,52,53]. Specifically, in IMPower133 and CASPIAN, the addition of atezolizumab and durvalumab, respectively, improved the OS by 2–3 months compared to chemotherapy alone. This led to the incorporation of IO into the standard of care for ES-SCLC. In all trials, PCI was permitted at the investigator’s discretion for all patients except in the CASPIAN trial, in which only the control arm could receive prophylactic cranial irradiation (8%). However, on closer analysis, the percentage of patients who received PCI in these trials was very limited, making the benefits/toxicity of PCI with IO difficult to interpret. The IMpower133 and CASPIAN trials demonstrated the potential for immunotherapy to delay intracranial relapses; however, they have faced challenges in conducting subgroup analyses comparing prophylactic cranial irradiation (PCI) use. This is due to the relatively small number of patients receiving PCI in both studies (10% in the IMpower133 and 8% in the control arm of CASPIAN) and the presence of missing data. PCI was not permitted in the experimental arm of CASPIAN.

A phase II STIMULI trial sought to evaluate the benefits of adding consolidation nivolumab with ipilimumab for patients who have not progressed following standard chemoradiation and PCI [54]. However, the study was closed prematurely due to slow accrual. It showed that there was no statistically significant improvement in PFS with immunotherapy compared with a placebo, and the benefits of PCI were not analyzable, as all patients received PCI; however, on further exploratory analysis, there was a statistically significant overall survival benefit in the subgroup of patients who had twice-daily radiation and received nivolumab and ipilimumab, which, while underpowered, raises the hypothesis that patients treated twice daily might benefit more. Two other randomized trials, NRG-LU005 and AdvanTIG-204, have also not shown a benefit from combining concurrent immunotherapy with thoracic chemoradiotherapy, but no further details regarding PCI have been provided [55,56].

The trial that changed the landscape regarding the role of IO in LS-SCLC was the phase III ADRIATIC trial, which evaluated consolidation IO with durvalumab with or without tremelimumab and demonstrated improved outcomes with consolidation immunotherapy [57]. In this trial, the disease stage and PCI were stratification factors. Notably, on subgroup analysis, patients receiving PCI in both arms had better outcomes than those who did not. Among the patients who underwent PCI, the three-year OS rates were 62.1% for patients in the durvalumab group compared with 56.5% in the placebo, with an HR of 0.75 (95% CI, 0.52–1.07). Among the patients who did not undergo PCI, the respective three-year OS rates were 50.2% in the durvalumab group and 37.3% in the placebo group, with an HR of 0.71 (95% CI, 0.51–0.99). The two-year PFS rates for patients who received PCI also favored durvalumab, at 54.6% in the durvalumab group and 38.5% in the placebo group. Among those who did not receive PCI, the two-year PFS rate was 37.1% in the durvalumab group and 29.3% in the placebo group, although this difference was not statistically significant. PCI in ADRIATIC was at the physician’s discretion, and the results need to be interpreted cautiously, as this may introduce a potential selection bias. So, for patients with LS-SCLC, definitive chemoradiation and PCI largely remain the standard of care in the setting of consolidative durvalumab.

Given the improved outcomes that these trials in both ES and LS-SCLC show, the addition of immunotherapy may influence the necessity and effectiveness of PCI in this patient population. To investigate these questions further regarding PCI, recent trials are ongoing to evaluate the efficacy of PCI in combination with immunotherapy and MRI surveillance.

## 7. Toxicity in PCI

Prophylactic cranial irradiation (PCI) causes a range of toxicities, both acute and long-term. Acute side effects include alopecia, fatigue, and raised intracranial tension and its corresponding symptoms, including headaches, nausea, and vomiting. These symptoms are often self-limiting or treatable with medications but can impact a patient’s quality of life during treatment. Typically, acute toxicities come within three months of treatment, with longer-term side effects emerging beyond the three-month mark.

Neurocognitive impairment is a long-term risk associated with PCI. Grosshans et al. demonstrated in a study that baseline neurocognitive impairment was seen in about 47% of SCLC patients before undergoing PCI [58]. However, it is also well recognized and should not be ignored that there are multiple factors other than PCI which can influence patients’ cognition. These are paraneoplastic syndromes, side effects from systemic treatment, anxiousness due to diagnoses of cancer, smoking history, etc.

Many studies have reported memory dysfunction as a treatment-related side effect, with both short-term and long-term memory effects. In a pooled secondary analysis of RTOG 0212 and RTOG 0214 (in the absence of HA), significant declines in recall and delayed recall were observed using the Hopkins Verbal Learning Test at both 6 and 12 months post-treatment [59]. These findings are further supported by retrospective data showing an increased risk of dementia in patients receiving PCI, as assessed using the Hasegawa Dementia Scale-Revised—42% compared to 5% among those who did not undergo the procedure [60]. Long-term cognitive decline was also found in a retrospective study of SCLC survivors treated with PCI, where nearly half of the patients demonstrated global cognitive worsening by studying gray and white matter density changes in MRIs and correlating them with cognitive deterioration scores; however, the sample size was small, including only 11 patients and patients who also received platinum-based chemotherapy [61]. Elderly patients, more than 70 years old, and especially patients with vascular comorbidities, were found to experience higher rates of neurotoxicity and cognitive decline after PCI [62]. In addition to cognitive changes, motor dysfunction has also been observed following PCI. In a small study of 40 patients, Nakahara et al. found gait disturbances to be more prevalent among those who underwent cranial irradiation, although only in older patients > 65 years. PCI treatment-related sequalae have meaningful implications for mobility and independence, particularly in older patients > 65 years [60].

Despite well-documented toxicities, some studies have found that PCI-induced neural changes are not uniform for all patients following PCI. For example, in the landmark Slotman trial, no statistically significant differences were found between the PCI and control groups in terms of role functioning (*p* = 0.17), cognitive functioning (*p* = 0.07), or emotional functioning (*p* = 0.18) [13]. Furthermore, a study by Grosshans et al. initially noted a transient decline in executive function (pre-PCI mean, 15.6 ± 11.5; post-PCI, 27.1 ± 17.6 [*p* = 0.008]) and language (pre-PCI mean, 33.8 ± 9.9; post-PCI, 31.0 ± 9.0 [*p* = 0.049]) [58]. However, after the study controlled for noncentral nervous system disease progression, the declines in executive function were no longer found to be significant. Together, these findings illustrate the complex and multifaceted toxicity profile associated with PCI as well as the cancer itself and systemic therapy.

There are potential endocrine effects from cranial irradiation, but the typical doses used for PCI are below the thresholds for pituitary hypothalamic damage. Growth hormone deficiency may be seen with doses <30 Gy, but our typical PCI dose fractionation regimens when converted to biologically equivalent doses are well below that constraint; therefore, the risk of clinically significant endocrine dysfunction in adults from PCI is low [63].

The mitigation of PCI treatment-related toxicity is important, as the treatments are known to cause neurocognitive, motor, and endocrine side effects. Memantine, an NMDA receptor antagonist, has been studied extensively to determine its role in the preservation of neurocognitive function. RTOG 0614 was a phase III trial randomizing patients receiving WBRT to receive or not to receive memantine. The results revealed a significant improvement in executive function, processing speed, and delayed recognition with memantine [64]. The use of hippocampal avoidance (HA) during cranial irradiation has been shown to reduce the deleterious neurocognitive changes associated with PCI, as discussed earlier. Combining hippocampal avoidance with the use of memantine further improves cognitive preservation. In a randomized phase III trial, patients treated with both HA-PCI and memantine found a significantly better preservation of cognitive function compared to those who received PCI alone [65]. Treatment scheduling has also been shown to improve neurological function for patients who underwent PCI. The use of lower fractional doses and avoiding concomitant chemotherapy have been shown to significantly reduce the incidence of neurotoxic effects, with the standard recommendation that chemotherapy and radiotherapy be scheduled separately [21]. Patient selection is also another factor when trying to mitigate treatment-related toxicity. Older adults, particularly those over 70 years of age, are at substantially higher risk for neurocognitive sequelae from PCI, especially if they have vascular comorbidities or early dementia syndromes [66]. In clinical practice, however, these patients are often underrepresented in trials due to the preclusion of comorbid conditions and poor performance status. The underrepresentation of elderly patients can potentially lead to an underestimation of toxicity risks in the clinic [62], and PCI may be approached with more caution in elderly patients.

## 8. The Evolving Role of SRS in SCLC Brain Metastasis Management

Historical data have estimated that 10–20% of patients with solid tumors do develop cranial metastases [67]. That number is expected to grow with increased surveillance and improved systemic therapies. Since the mid-20th century, WBRT has been most widely offered to patients with multiple brain metastases. There was little focus on WBRT toxicities such as cognitive deterioration, as the primary focus was on survival, which was very poor [68]. RTOG 9508 established the role of SRS in limited brain metastases, but it excluded SCLC patients [69].

In a phase III EORTC study, patients with up to three brain metastases of solid tumors (excluding SCLC) who were treated with surgical excision or SRS were randomized to either adjuvant WBRT (30 Gy/10 Fx) or observation. The results showed that OS was similar in the WBRT and observation arms [70]. WBRT reduced the relapse rate both at initial sites and at new sites. Several other studies of solid tumor patients with cranial metastases were conducted while excluding SCLC patients, which showed that the addition of WBRT to SRS leads to greater learning, memory decline, and cognitive deterioration [71,72].

The FIRE-SCLC study evaluated first-line SRS vs. WBRT for SCLC. The SRS median overall survival was 8.5 months, the median time to CNS progression was 8.1 months, and the median CNS progression-free survival was 5 months. In a propensity score-matched analysis comparing the outcomes of WBRT versus SRS, WBRT was associated with an improved time to CNS progression, indicating a reduced risk of CNS progression. However, WBRT did not improve OS, 6.5 vs. 5.2 months, or CNS PFS, 4.0 vs. 3.8 months [73]. A large meta-analysis that included nine observational studies with 1638 patients assessed the upfront SRS effectiveness for SCLC brain metastases in comparison to WBRT. The median OS was found to be 8.3 months [74]. The projected OS for 6, 12, 18, and 24 months compared with SRS with WBRT was 67% vs. 57%, 39% vs. 29%, 22% vs. 15%, and 15% vs. 9%, favoring SRS (*p* < 0.001). High local control rates were observed (93%), and the distant brain failure rate at 12 months was 41%. This meta-analysis showed that, in SCLC with limited brain metastases, upfront SRS improved lesion control and survival outcomes. Another review and meta-analysis by Gaebe et al. comparing SRS vs. WBRT for intracranial metastasis in SCLC demonstrated no significant difference in survival with SRS vs. WBRT [75].

The CROSS-FIRE study by Rusthoven et al. retrospectively analyzed the results of SRS for both NSCLC and SCLC. OS was significantly better for NSCLC patients (*p* < 0.001). However, there was no significant difference in CNS progression in the two groups in matched cohorts. Similarly, no difference in neurological mortality was observed between groups, and both groups had a similar number of lesions at the time of CNS progression [76]. An ongoing single-center prospective randomized study (Encephalon Trial) is comparing neurocognitive function. Patients will be randomized to receive either SRS for all brain metastases (up to ten lesions) or WBRT. Another retrospective study by Wang et al. evaluated SCLC patients who received SRS. Of the 70 patients treated, more than half had prior WBRT. There was no difference in survival between the patients treated with prior WBRT and then SRS compared to SRS alone. However, patients who were treated with SRS alone had an improved distant brain failure if they had five or fewer brain metastases (*p* < 0.040) [77].

Further studies are being conducted to prospectively compare the benefits of SRS versus HA-WBRT with memantine, such as the current ongoing trial (NRG CC09) that is enrolling any patient with SCLC with 1–10 mets and assessing the time to cognitive failure between the two modalities [NCT04804644]. The evidence from the prospective phase II trial from Harvard shows promising results, as patients treated with SRS were found to have lower rates of neurologic death when compared to WBRT [78]. This may lead to a shift towards incorporating SRS as a salvage option for SCLC patients undergoing MRI surveillance after a risk–benefit assessment.

## 9. Ongoing Trials

Most ongoing trials for PCI are now incorporating strategies to minimize the neurotoxicity of PCI, while at the same time evaluating the efficacy of PCI in the current immunotherapy era. NRG CC 009 is evaluating whether SRS is a more effective treatment option than hippocampal avoidance whole-brain radiotherapy (HA-WBRT) plus memantine in preventing cognitive decline in patients with brain metastases from SCLC.

A UK-based EORTC PRIMA lung trial and the MAVERICK (SWOG1827) trial are both non-inferiority trials currently investigating MRI surveillance with early treatment and MRI surveillance with PCI in patients with limited- and extensive-stage SCLC [79]. These trials allow the utilization of hippocampal avoidance techniques with OS as the primary endpoint, and secondary objectives include cognitive failure-free survival, quality of life, and toxicities associated with PCI and IO. The MAVERICK trial has pre-randomization stratification, which includes LS vs. ES, the use of IO, and PS 0-1 vs. 2. Stratification with the use of immunotherapy delivery can aid in the analysis of the benefits and interaction of PCI and IO. The results of these trials may shed more light on the optimal management strategy for SCLC regarding PCI in the current realm of MRI screening/surveillance and IO.

## 10. Future Directions

Risk stratification and patient selection are crucial in the management of CNS disease in patients with SCLC, as choosing between PCI, MRI surveillance, and SRS in the modern era where systemic therapy options with immunotherapy and novel targeted therapies with possible CNS penetration are available is still an area which needs further exploration. A recent study analyzed circulating tumor DNA (ctDNA) in 33 patients with extensive-stage SCLC and showed that ctDNA levels and response patterns correlate strongly with clinical response and survival outcomes [80]. Using ctDNa to refine and personalize the use of consolidation IO has been underway in China, and the results showed that early ctDNA detection after induction chemotherapy can help identify patients who are more likely to benefit from consolidation immunotherapy [81]. Prospective trials using ctDNA as an integral biomarker for therapeutic selection should be considered in SCLC to optimally manage CNS disease. Additionally, emerging targeted agents such as tarlatamab, a CD3/DLL3 bispecific T-cell engager, have shown encouraging intracranial activity. Early reports indicate an approximately 90% clinical response or disease stability in patients with previously untreated brain metastases [82].

## 11. Conclusions

To summarize, PCI remains the standard of care for patients with LS-SCLC who respond to primary treatment. PCI should be considered in all LS-SCLCs except stage I patients who undergo a complete removal of the tumor. In patients with ES-SCLC who pledge to comply with regular follow-up MRI to detect brain metastases, PCI can be avoided. Also, with the recent immunotherapy trials in ES-SCLC not considering PCI as a relevant treatment component in their study protocols, and with a very small proportion of recruited patients receiving PCI, its role has become blurrier. For ES-SCLC, it is suggested to have an individual discussion with each patient, weighing the pros and cons of each treatment option and then making an informed decision. Perhaps we will have a clearer picture of the role of PCI in ES SCLC after the results of the newer studies, which are combining PCI with IO. Also, HA has been studied sparingly in SCLC due to the presumed risk of failure in the spared brain, but there have been some promising results, and probably future bigger studies will give us clearer picture of the role of HA in SCLC. Even though the newer immune checkpoint inhibitors for the treatment of SCLC have shown modest benefits in terms of survival, they have still drawn significant attention; however, at the same time, we should not overlook the benefits of treatments like PCI, which has shown survival benefits and has been the standard of care for a long time, with improvements in treatment techniques over time to minimize the toxicities. Although initially PCI was thought to delay the occurrence of brain metastasis and its associated complications, now, with the early detection of brain metastases with serial MRIs of the brain and the availability of salvage options with surgery and SRS, its role is being reappraised. Thus, while PCI remains a cornerstone in the management of selected patients with SCLC, its role must be individualized, integrating the disease stage, patient preferences, emerging technologies, and long-term survivorship considerations.

## Figures and Tables

**Table 1:** ESTRO, ASTRO, and NCCN guidelines for PCI in SCLC.

Organization	PCI in SCLC
European Society for Radiotherapy and Oncology (ESTRO)	PCI and thoracic radiation therapy should be utilized in patients with resected SCLC and positive nodes. Not recommended by most experts to utilize PCI or thoracic RT in elderly patients with resected node-negative SCLC [3].
American Society for Radiation Oncology (ASTRO)	In LS-SCLC, PCI is strongly recommended for patients with stage II or III who respond to chemoradiation. It is conditionally not recommended for patients with stage I disease and should be a shared decision with patients at higher risk of neurocognitive toxicities. For ES-SCLC, it is highly recommended that a consultation be placed with a radiation oncologist to discuss PCI versus brain MRI surveillance [4].
National Comprehensive Cancer Network (NCCN)	In LS-SCLC patients who respond well to initial therapy, PCI decreases brain metastases and increases overall survival rate. Guidelines note PCI may have benefits in patients with pathological staging IIB or III after complete resection. The benefits of PCI are unknown in patients pathologically staged I-IIA (T1-2 N0 M0) SCLC. In ES-SCLC for patients responsive to systemic therapy, PCI decreases brain metastases. Brain MRI surveillance is recommended for all patients regardless of PCI status (NCCN Clinical Practice Guidelines in Oncology. Small Cell Lung Cancer, 2025) [5].

**Table 2:** Immunotherapy agents in SCLC.

IO Drug	Class	Landmark Trial	Year	Indication	% Receiving PCI
Nivolumab + Ipilimumab	PD-1 inhibitor + CTLA-4 inhibitor	CheckMate 032	2016	Metastatic SCLC; third-line	N/A
Atezolizumab	PD-L1 inhibitor	Impower133	2018	ES-SCLC; first-line	~11% patients in each arm
Durvalumab	PD-L1 inhibitor	CASPIAN	2020	ES-SCLC; first-line	~8% patients in control arm
Durvalumab	PD-L1 inhibitor	ADRIATIC	2024	LS-SCLC; adjuvant	~54% total

## Data Availability

No new data were created or analyzed in this study. Data sharing is not applicable to this article.
